# “Pseudo aortoiliac bifurcation” leading to significant plaque shifting in the endovascular treatment of an aortoiliac bifurcation lesion: a case report

**DOI:** 10.1186/s12872-017-0614-2

**Published:** 2017-07-04

**Authors:** Yoshito Kadoya, Tsuneaki Kenzaka, Daisuke Naito, Kan Zen, Satoaki Matoba

**Affiliations:** 1 Department of Cardiovascular Medicine, Graduate School of Medical Science, Kyoto Prefectural University of Medicine, 465 Kajii-cho, Kawaramachi-Hirokoji, Kamigyo-ku, Kyoto, 602-8566 Japan; 2 Division of Community Medicine and Career Development, Kobe University Graduate School of Medicine, Kobe, Japan; 3 Department of Cardiology, Kyoto City Hospital, Kyoto, Japan

**Keywords:** Endovascular treatment, Complication, Plaque shifting, Case report

## Abstract

**Background:**

Plaque shifting is a serious complication of endovascular treatment (EVT) for aortoiliac bifurcation lesions. It is challenging to predict the occurrence of unfavorable plaque shifting correctly**.**

**Case presentation:**

We report the case of an 88-year-old Japanese woman who experienced constant pain at rest in her left leg. The ankle-brachial pressure index of her left leg was 0.57. Computed tomography (CT) angiography revealed severe stenosis of the left common iliac artery (CIA) and total occlusion of the left external iliac artery (EIA). We diagnosed the patient with acute exacerbation of a chronic limb ischemia and administered endovascular treatment (EVT) to treat the left CIA and EIA. The results of initial angiography agreed with those of CT angiography. After placing a self-expandable stent for the left CIA lesion, significant unfavorable plaque shifting occurred. From a comparison between pre- and post-stenting angiography, we realized that the plaque protrusion into the terminal aorta had formed a “pseudo aortoiliac bifurcation” that was situated more proximally compared to the true bifurcation. We had incorrectly assessed the height of the aortoiliac bifurcation and exact plaque position and had underestimated the risk of plaque shifting because of this misunderstanding. The patient ultimately developed fatal cholesterol embolization after EVT.

**Conclusions:**

Plaque protrusion into the terminal aorta can form a “pseudo aortoiliac bifurcation”, causing the wrong estimation of the height of the aortoiliac bifurcation; “angiographically”, the highest point is not always the true bifurcation. Careful assessment of initial angiography to detect the true aortoiliac bifurcation and exact plaque position is essential to avoid unfavorable plaque shifting.

**Electronic supplementary material:**

The online version of this article (doi:10.1186/s12872-017-0614-2) contains supplementary material, which is available to authorized users.

## Background

Endovascular treatment (EVT) at the level of the aortic bifurcation has been widely performed in recent years. The EVT strategy for aortoiliac bifurcation lesions has been discussed in the literature [1–3]. The most important concern with regard to these lesions is plaque shifting or embolization of the contralateral vessel. To avoid these serious complications, the kissing balloon technique, in which balloons are simultaneously positioned across both limbs of the aortic bifurcation and inflated in unison, has been reported to be effective [2]. Later as stents became widely used, the kissing stent technique was adapted instead of the kissing balloon technique, especially in the case of dissection, thrombosis, or significant residual stenosis [3]. However, it is still challenging to predict the occurrence of unfavorable plaque shifting correctly. Here, we report a case of significant unfavorable plaque shifting after stenting for an aortoiliac bifurcation lesion, caused by a “pseudo aortoiliac bifurcation” formed by plaque protrusion into the terminal aorta.

## Case presentation

**Fig. 1:**
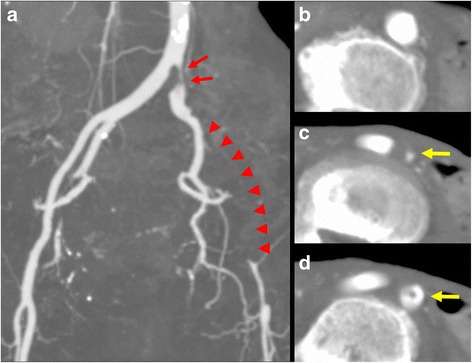
**a** Computed tomography angiography revealing severe stenosis of the left common iliac artery (*red arrows*) and total occlusion of the left external iliac artery (*arrowheads*). **b-d** Axial scan of the bifurcation showing a non-calcified plaque existing at the ostium of left CIA (*yellow arrows*)

**Fig. 2:**
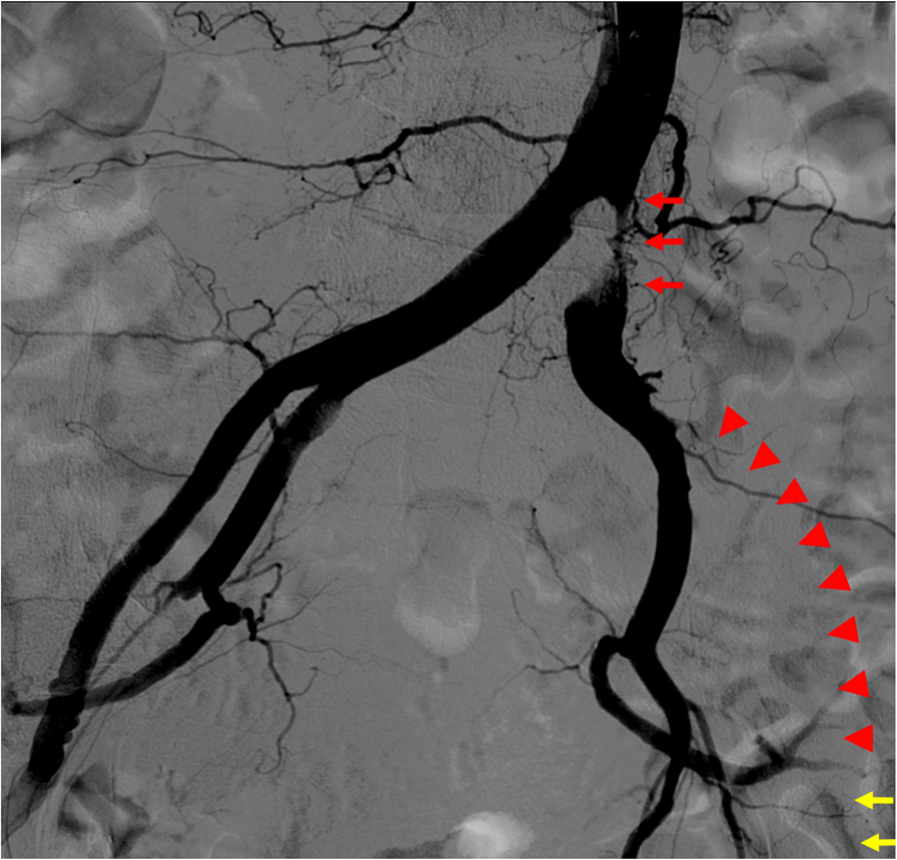
Initial angiogram. Initial angiography showed severe stenosis of the left common iliac artery (*red arrows*) and total occlusion of the left external iliac artery (*arrowheads*). The common femoral artery was delayed-enhanced via collateral circulation (*yellow arrows*)

**Fig. 3:**
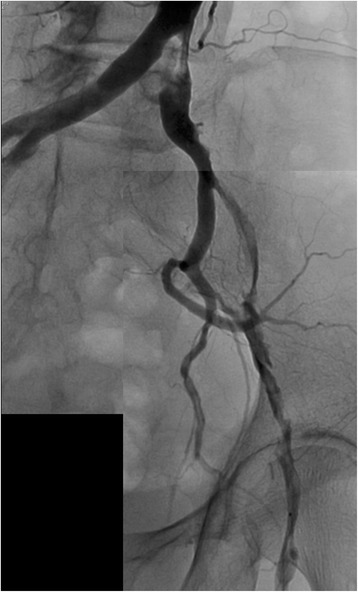
Angiography showing good antegrade blood flow after the pre-dilatation

**Fig. 4:**
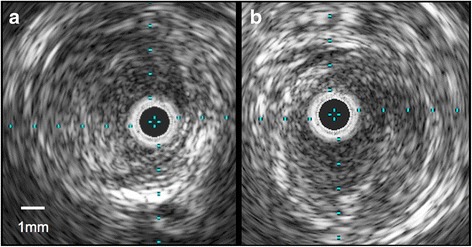
Intravascular ultrasound image. IVUS imaging in the external iliac lesion showing the wire passing through the true lumen and the mixed plaque with no calcification. (**a** proximal site of the left external iliac artery, (**b**) distal site of the left external iliac artery)

**Fig. 5:**
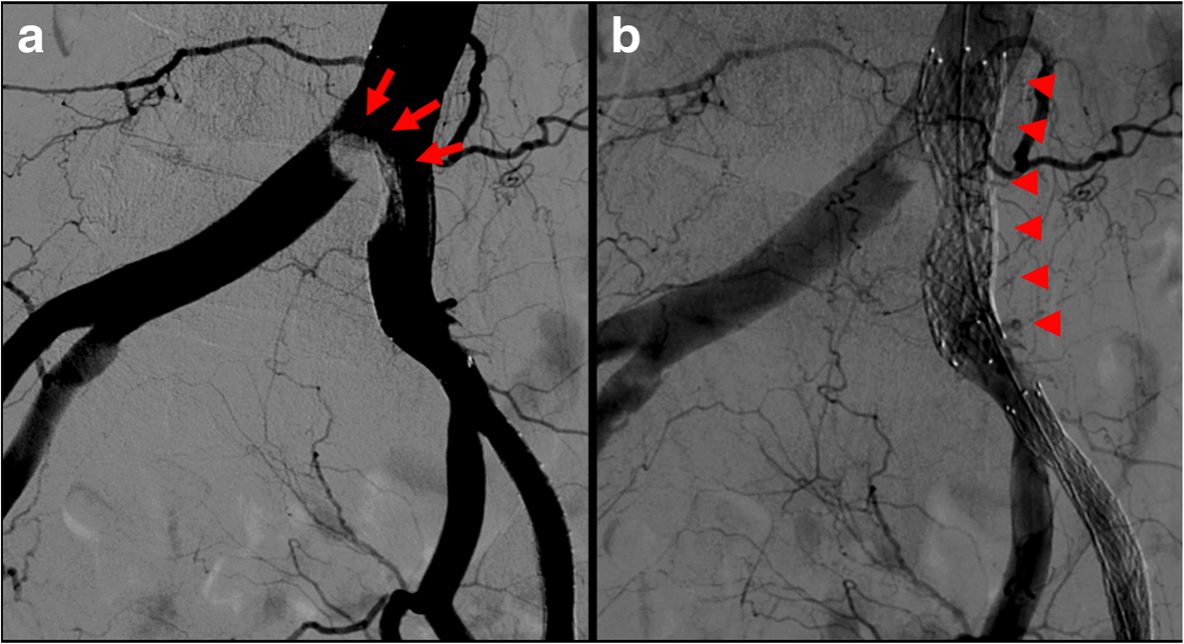
Angiogram after stent placement.** a** Angiography after stent placement showed significant plaque shifting to the right common iliac artery (*arrows*). **b** The stent in the left common iliac artery completely crossed over the contralateral common iliac artery (*arrowheads*)



An 88-year-old Japanese woman with bacterial pneumonia was admitted to our hospital. Her medical history included angina pectoris and nontuberculous mycobacterial pulmonary infection. She had no history of atrial fibrillation. She occasionally felt pain at rest in her left leg during hospitalization. Pneumonia resolved with antibiotic therapy. On the 23rd day of hospitalization, she complained of constant pain at rest and cyanosis in the left leg. Two days later, the pain and skin color worsened. Her body temperature was 36.6 °C; blood pressure, 150/70 mmHg; regular pulse rate, 80 beats/min; and oxygen saturation, 93% (without oxygen administration). The ankle-brachial pressure index of the left leg was 0.57. Myogenic enzymes and lactic acid were not elevated in laboratory findings, and the left lower limb had not become necrosed. Computed tomography (CT) angiography revealed severe stenosis of the left common iliac artery (CIA) and total occlusion of the left external iliac artery (EIA) (Fig. 1a). As for the CIA lesion, the plaque seemed to exist at the ostium of the CIA (Fig. 1b-d). The arteries below the knee were also severely stenosed or occluded. We made a diagnosis of acute exacerbation of chronic limb ischemia and considered that the possibility of acute embolization was relatively low. We discussed the treatment strategy, including optimal medical therapy or surgical bypass grafts. Considering the patient’s age, severe symptoms, and general condition, we thought the endovascular approach would be more suitable. We administered EVT to treat the left CIA and EIA lesion, in addition to the medical treatment comprising heparin, aspirin, and alprostadil. The results of initial angiography agreed with those of CT angiography (Fig. 2), and we planned to place stents for both CIA and EIA lesions. Since the left common femoral artery (CFA) had moderate to severe stenosis and the crossover approach via the right CFA seemed to be difficult because of the left CIA lesion, we inserted a 6-Fr Sheathless PV® guiding catheter (Asahi Intecc, Japan) into the terminal aorta via the left brachial artery. We attempted antegrade wiring using a 0.018-in. wire with intravascular ultrasound (IVUS), changing wires several times. We finally passed a 0.014-in. wire into the left CFA. We performed pre-dilatation with a 4-mm balloon for both CIA and EIA lesions, and thrombus aspiration for the EIA lesion using a 6-Fr straight guiding catheter but no thrombus was aspirated. Subsequently, angiography showed good antegrade blood flow (Fig. 3). To simplify the adjustment of the stent deployment position, especially the stent proximal edge, we switched to the left CFA approach; we re-punctured the left CFA and passed the wire into the aorta. IVUS in the EIA lesion showed that the wire passed through the true lumen and the lesion consisted chiefly of mixed plaque with no calcification (Fig. 4). We did not perform IVUS for the CIA lesion. We first performed single-stenting using a self-expandable stent, an Epic® stent (120 × 40 mm) (Boston Scientific, USA), for the left CIA lesion, and then placed two other Epic® stents (90 × 100 mm and 80 × 40 mm, respectively) at the EIA lesion. After post-dilatation with a 5-mm balloon for each lesion, angiography showed significant plaque shifting to the right CIA (Fig. 5a, Additional file 1: Video S1). Moreover, the stent in the left CIA completely crossed over the contralateral CIA (Fig. 5b, Additional file 1: Video S1). We inserted a 6-Fr sheath via the right CFA and performed an IVUS for the shifted plaque. A large mobile plaque with a high risk of distal embolization was observed. We considered that it would be challenging to implant an additional stent in the right CIA to cover the plaque. Therefore, we performed long balloon inflation with an 8-mm balloon to compress and stabilize the shifted plaque. The final angiography showed good blood flow in both lower limbs with no evidence of distal embolization. Since there was no flow limitation or pressure gradient across the shifted plaque, we completed the procedure. On the day after EVT, the patient developed livedo reticularis on both lower limbs, with a high inflammatory response and worsening renal function. Although eosinophilia was not observed, we considered that the cause of death was cholesterol embolization based on this clinical course. Eventually, the patient died of multiple organ failure 3 days after EVT despite treatment.

**Additional file 1: Video S1.** Angiography after stent placement showed significant plaque shifting to right common iliac artery (arrows). (MP4 2203 kb)


## Discussion

We present a case of significant plaque shifting after stenting in an aortoiliac bifurcation lesion. Because of the “pseudo aortoiliac bifurcation” formed by plaque protrusion into the terminal aorta, we incorrectly assessed the true aortoiliac bifurcation and exact plaque position and underestimated the risk of plaque shifting. To our knowledge, this is the first report of this phenomenon.

**Fig. 6:**
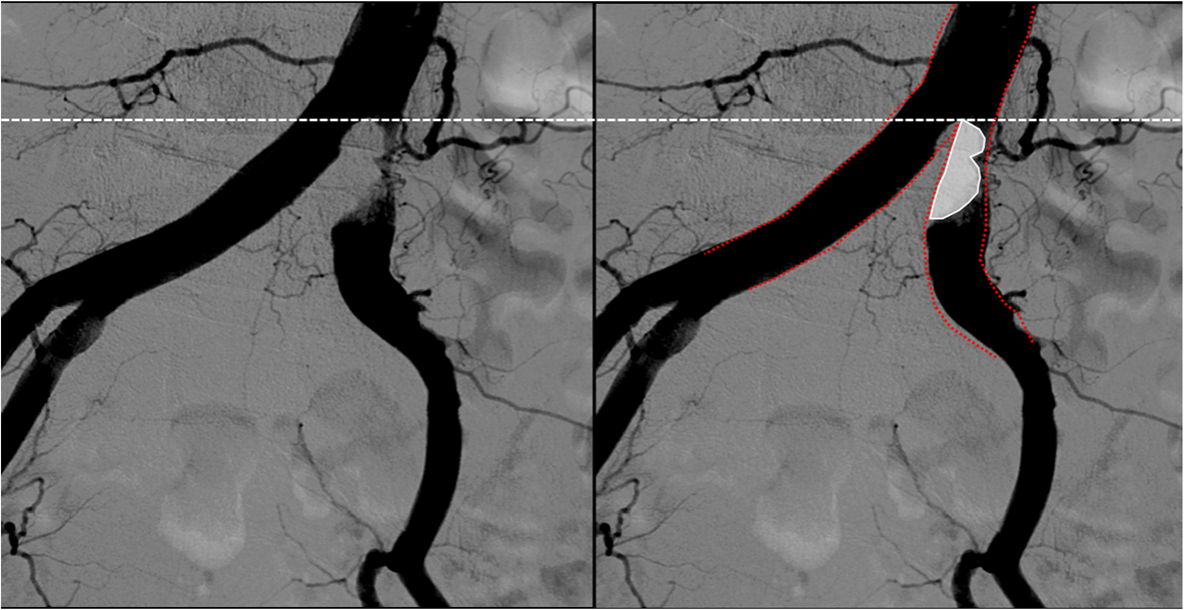
Our initial impression regarding the height of aortoiliac bifurcation and plaque position. We thought that “angiographically”, the highest point was the aortoiliac bifurcation and the plaque existed only in the left CIA. (*left side*: pre-stenting angiography, *right side*: post-stenting angiography, *white dash line*: the height of the aortoiliac bifurcation according to our initial impression, red dash line: the vessel *wall line* of aorta and bilateral iliac artery according to our initial impression, *white line*: plaque position according to our initial impression)

**Fig. 7:**
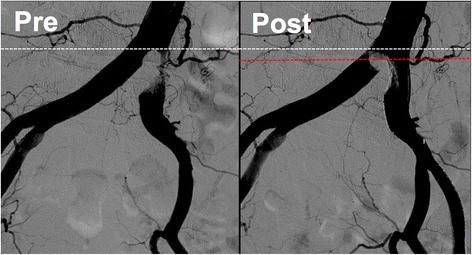
Comparison between pre-stenting and post-stenting angiography. Careful comparison showed that the height of the aortoiliac bifurcation had changed as against our initial impression; the height of the aortoiliac bifurcation moved more distally after stenting. (*left side*: pre-stenting angiography, *right side*: post-stenting angiography, *white dash line*: the height of the aortoiliac bifurcation according to our initial impression, *red dash line*: the height of the true aortoiliac bifurcation revealed by the post-stenting angiography)

**Fig. 8:**
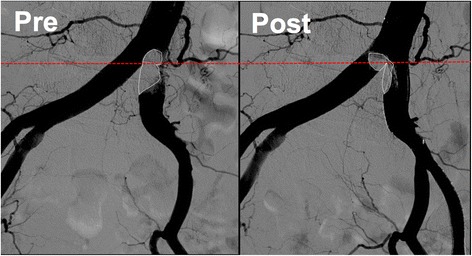
A “pseudo aortoiliac bifurcation” had been formed by the plaque protrusion into the terminal aorta (*left side*: pre-stenting angiography, *right side*: post-stenting angiography, white *dash line*: the exact plaque position, *red dash line*: the height of the true aortoiliac bifurcation)

**Fig. 9:**
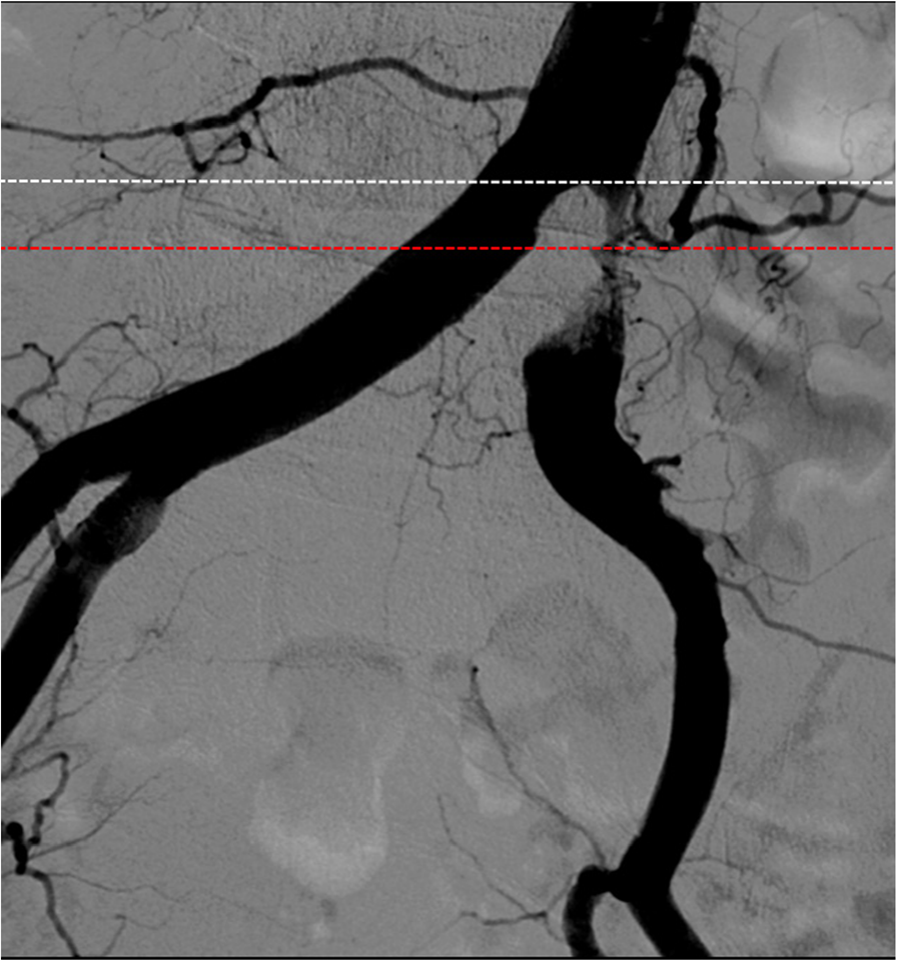
The true aortoiliac bifurcation existed more distally than “angiographically” the highest point. (*white dash line*: initially misunderstand about the aortoiliac bifurcation. *Red dash line*: true aortoiliac bifurcation)

In the present case, we initially thought that “angiographically”, the highest point was the aortoiliac bifurcation and the plaque existed only in the left CIA (Fig. 6). We planned to perform stenting at the ostium of the left CIA with minimum protrusion into the aorta without obstructing the entry to the contralateral CIA. However, post-stenting angiography showed that the height of the aortoiliac bifurcation had changed, moving more distally (Fig. 7). From a careful comparison between the pre- and post-stenting angiography, we realized that we had incorrectly assessed the aortoiliac bifurcation and exact plaque position; a large plaque had protruded into the terminal aorta and formed the “pseudo aortoiliac bifurcation” (Fig. 8). The true aortoiliac bifurcation existed more distally than the “angiographically” highest point (Fig. 9). We underestimated the risk of plaque shifting because of this misunderstanding.

On a retrospective review of the initial angiography, the vessel wall of the “pseudo aortoiliac bifurcation” was slightly unnatural. If we had assessed the initial angiography more carefully and noticed the plaque protrusion and the “pseudo aortoiliac bifurcation” beforehand, we would have performed the kissing balloon technique or kissing stent technique if dissection or residual stenosis had occurred. However, our initial evaluation that the plaque had not protruded into the aortoiliac bifurcation was wrong, leading to the single-stent strategy. We should have realized that the plaque could protrude into the terminal aorta and form the “pseudo aortoiliac bifurcation”, because “angiographically”, the highest point is not always the true aortoiliac bifurcation. Regarding the bailout method for the plaque shifting, there are no definitive methods for this kind of situation. In fact, we did not discover a better way to manage this complication. Thus, it is important to prevent unfavorable plaque shifting as much as possible.

## Conclusions

We described a case of significant plaque shifting due to a “pseudo aortoiliac bifurcation” formed by plaque protrusion into the terminal aorta. Careful assessment of initial angiography is essential for detecting the true aortoiliac bifurcation and exact plaque position in order to prevent unfavorable plaque shifting.

## Abbreviations

CFA: Common femoral artery
CIA: Common iliac artery
CT: Computed tomography
EIA: External iliac artery
EVT: Endovascular treatment
IVUS: Intravascular ultrasound
